# Deposition, Dietary Exposure and Human Health Risks of Heavy Metals in Mechanically Milled Maize Flours in Mbarara City, Uganda

**DOI:** 10.3390/jox13030022

**Published:** 2023-06-26

**Authors:** Herbert Kariitu Mugume, Denis Byamugisha, Timothy Omara, Emmanuel Ntambi

**Affiliations:** 1 Department of Chemistry, Faculty of Science, Mbarara University of Science and Technology, Mbarara P.O. Box 1410, Uganda; 2 Food Safety Laboratories, Chemistry Division, Testing Department, Standards Directorate, Uganda National Bureau of Standards, Bweyogerere Industrial and Business Park, Kampala P.O. Box 6329, Uganda; 3 Institute of Chemistry of Renewable Resources, Department of Chemistry, University of Natural Resources and Life Sciences, Vienna (BOKU), Konrad-Lorenz-Straße 24, 3430 Tulln, Austria

**Keywords:** toxic metals, hammer mills, maize, estimated daily intake, cancer risk, hazard quotient

## Abstract

Consumption of maize and maize-based products contributes a significant percentage to the total food energy intake in Uganda. However, the production of maize-derived foodstuffs is performed traditionally or by small- and medium-scale processors using different processing techniques. This can lead to differences in the quality of these products from processors, raising food safety concerns. In this study, the effects of mechanical processing (milling) methods on deposition of heavy metals into milled maize flour and the associated consumption health risks were assessed. Atomic absorption spectrophotometry was used to quantitatively establish the concentration of iron (Fe), manganese (Mn), zinc (Zn), cadmium (Cd), lead (Pb), chromium (Cr), copper (Cu), cobalt (Co) and nickel (Ni) in 100 samples of maize milled using a wooden mortar (*n* = 2), a metallic mortar (*n* = 2), diesel engine−powered mills (*n* = 48) and electric motor−powered mills (*n* = 48). Results showed that the mean concentrations of heavy metals in mg/kg were Fe (11.60–34.45), Cu (0.50–8.10), Ni (0.50–1.60), Mn (0.70–25.40), Zn (4.40–15.90), Pb (0.53–10.20), Cd (0.51–0.85), Cr (0.50–1.53) and Co (0.50–1.51). The highest concentrations were found in flour milled using a traditional metallic mortar while the lowest levels were in those samples milled using a wooden mortar. The Fe, Pb and Cd contents of flours produced using the metallic mortar and some commercial mills was found to be higher than the permissible limits set by WHO/FAO. Human health risk assessment showed that there are potential carcinogenic health risks from adults’ intake of heavy metals in maize flour milled using a metallic mortar. Therefore, processing of maize flour needs to be monitored by the relevant statutory bodies in Uganda to minimize the possibility of heavy metal contamination of food products and animal feeds.

## 1. Introduction

Food is nutritionally considered to be the primary source of essential human micronutrients such as copper (Cu), cobalt (Co), nickel (Ni), iron (Fe), molybdenum (Mo), manganese (Mn), selenium (Se) and zinc (Zn) [1,2]. However, dietary intake of contaminated foods can also be a major route for entry of toxic chemicals into humans. This is particularly inevitable where food safety and quality are not rigorously monitored [2,3]. Maintaining food safety has remained a global challenge with public health implications and huge socioeconomic consequences. For example, conservative estimates provided by the WHO Foodborne Disease Burden Epidemiology Reference Group indicate that ingestion of unsafe food causes at least 600 million global incidences of foodborne diseases and 420,000 mortalities which can be equated to an annual loss of 33 million disability-adjusted life years [4,5]. More precise examples include hepatocellular carcinoma and mortalities due to the consumption of mycotoxin-contaminated maize in Kenya [6] and the tragic incidence of Minamata disease linked to the ingestion of methylmercury-contaminated aquatic foods [7].

Food safety hazards are of biological, chemical and physical nature and may be present at the same time in a given food matrix [8]. They may also interact with each other, giving an amplified toxicity or injurious outcomes. Unlike other food hazards, chemical food hazards tend to have long-lasting effects because some of them are probable carcinogens [9]. In this context, heavy metals (HeMs) have been one of the critical parameters monitored in food and food products globally. HeMs are by definition those chemical elements with high molecular weights and specific gravity (i.e., at least five times greater than that of water) [10]. This broad definition leads to the listing of Cu, Fe, Zn, Ni, lead (Pb), vanadium, arsenic (As), cadmium (Cd), tin (Sn), chromium (Cr), Co, mercury, Mo, Ni, strontium (Sr) and titanium (Ti) as HeMs [11]. Though they are known to be toxic, the severity of the toxic effects from excessive intake of HeMs is influenced by valence states, exposure routes and duration, bioavailability, chemical forms and the ingested quantity of the metal(s), as well as the nutritional status, age and sex of the intoxicated organism [12].

Dietary exposure to HeMs has been extensively studied. However, most studies do not pay attention to potential introduction of these contaminants during food processing. A couple of empirical studies give credit that milling of cereals (such as millet, sorghum and maize) expose consumers to leached HeMs [13,14,15,16,17,18,19,20,21]. In Uganda, the per capita maize consumption is approximately 28 to 125 kg per annum [22], and maize is the third most important food in terms of caloric intake after plantain and cassava. Thus, consumption of maize and its products contribute more than 11% of the caloric intake [23,24,25] and up to 50% of the caloric intake in schools and tertiary institutions [26]. The production of maize-derived foodstuffs in Uganda is performed traditionally or by small- and medium-scale processors using different processing techniques. Of these, dry mechanical milling using non-food grade hammer mills contributes the largest percentage [27,28,29]. This can lead to differences in the quality of maize products from different processors, raising food safety concerns. Some studies have indicated that maize grown in Uganda is contaminated with HeMs [30,31,32]. Only one study has quantified HeMs in maize flour from Kampala, Uganda [33], but it did not take into consideration whether or not the milling process could have contributed to the overall concentration of the metals reported. This study was an attempt to establish the effects of mechanical processing methods (milling) on HeM contamination of milled maize consumed in Mbarara City, Western Uganda. A human health risk assessment model was used to establish if there are any potential carcinogenic and non-cancer risks that could arise from consumption of maize milled using the different mechanical methods.

## 2. Materials and Methods

### 2.1. Study Context and Geographical Coverage

**Figure 1:**
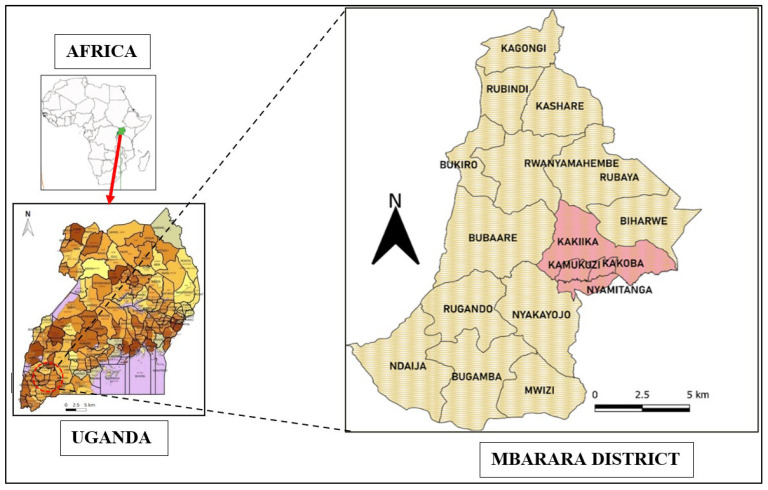
Map of Mbarara District showing the location of Mbarara City boroughs, Western Uganda where milling experiments were done.

**Figure 2:**
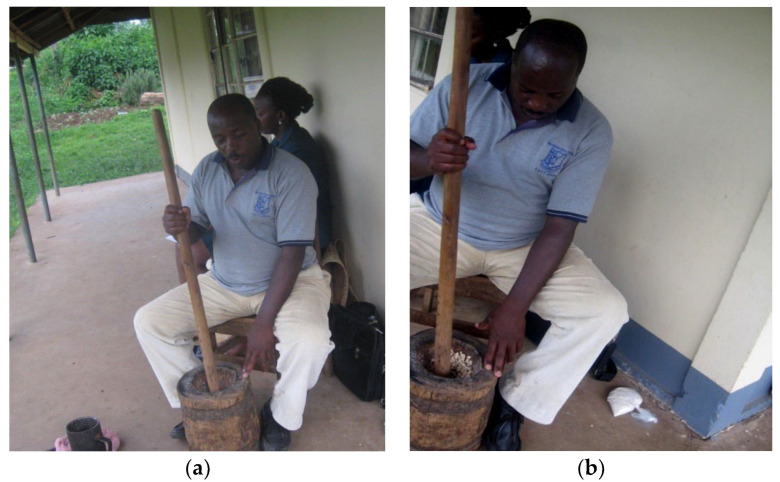
Traditional maize milling in Mbarara City, Western Uganda (**a**) wooden mortar and pestle. A metallic mortar is shown in the left foreground, and (**b**) grinding of maize grain in a traditional wooden mortar.

The study was limited to the quantification of HeMs (Fe, Mn, Zn, Cd, Pb, Cr, Cu, Co and Ni) in maize flour from Mbarara City, Western Uganda (Figure 1). The HeMs were quantified from the maize flour processed using wooden and metallic mortars (Figure 2) and diesel and electric motor−powered milling machines. For the diesel and electric motor−driven milling machines, the focus was on hammer-type mills. The area chosen for the study lies in the Mbarara district, which is about 260 km from Kampala, the capital city and central business district of Uganda. The population of Mbarara district is estimated at 69,208 people, and these people live in different administrative units of the Kamukuzi, Kakoba and Nyamitanga divisions. Various institutions such as Mbarara Municipal Primary School, Mbarara Junior School, Mbarara High School, Mary Hill High School and Ntare School, as well as Tertiary institutions (Mbarara University of Science and Technology−MUST, Makerere University Business School, Bishop Stuart University and Uganda Martyrs University) in Mbarara feed their students maize meal (porridge and posho). The maize grain mills in the city are also used to process maize into flour that is consumed in the surrounding sub-counties of the Mbarara District and the surrounding districts of Isingiro, Sheema, Bushenyi and Kiruhura.

### 2.2. Chemicals and Reagents

All reagents and chemicals used in this study were of high analytical purity (>95%; supplied by Merck (Rahway, NJ, USA) or Sigma Aldrich (Saint Louis, MO, USA), and were therefore used in the analytical work without prior purification. Unless otherwise indicated, all solutions were prepared using double distilled water.

### 2.3. Study Design

Dry maize grains (50 kg) were purchased from rural farmers in Kamwenge Sub County, Kamwenge District, Western Uganda. This is an agricultural area with a limited number of automobiles and no heavy industries that can pollute the environment via aerial deposition. In this area, farmers depend on the natural fertility of soils for growing their crops; thus, there is limited pollution through the use of synthetic fertilizers. Maize grains from this area are bought by the business community from Mbarara city who later transport, mill and sell the maize flour within the city.

Measured grains (1 kg) were washed and rinsed with distilled water, spread on a plastic sheet to dry indoors to reduce possible aerial pollution (deposition of HeMs). The clean grains (0.5 kg) were pounded into flour using a wooden mortar and pestle without any metallic contact. Similarly, another sample of the washed grains (0.5 kg) was pounded into flour using a metallic mortar and pestle. The two samples generated were used as controls. The remaining grains were milled into flour using selected grain milling machines in Mbarara city, Western Uganda.

The maize flour samples for laboratory analysis were collected between the months of December 2022 and March 2023. These were from four different diesel engine and electric powered hammer-type mills. The diesel mills were labeled A, B, C and D while the electric mills were coded E, F, G and H. The location of the mills was as follows: A and E in the Kamukuzi division, B and F in the Kakoba division, C and G in the Nyamitanga division and H and D in the Makenke division.

Three samples were collected in polythene bags from each of the mills (A, B, C, D, E, F, G and H) at two different intervals during each milling phase, that is, within the first 5 min when the flour first exits on starting and the second interval of sampling was when the flour would be exiting before milling is stopped. A total of 48 samples was collected from diesel engine-powered mills. Similarly, 48 flour samples were collected from the electric motor-powered mills. Two flour samples each were collected for maize processed using wooden and metallic mortars and pestles. Therefore, a total of one hundred (100) maize flour samples was collected and kept in airtight plastic buckets at room temperature in the Chemistry laboratory at the Department of Chemistry, Mbarara University of Science and Technology, Mbarara, Uganda.

### 2.4. Sample Preparation and Analysis

Analytical flour samples were obtained from pooled maize flour samples using the cone and quarter method. Each analytical sample (3 g) from the cone and quarter method was put on a porcelain crucible and dried in the oven for 24 h at 85 °C. The samples from the oven were removed and cooled in a desiccator to avoid reabsorption of moisture for dry basis analysis. Measured 2.0 ± 0.1 g of the samples were transferred into 250 mL beakers and cold digested with 100 mL of perchloric acid/nitric acid mixture (1:3 *v*/*v*) in a fumehood for 30 min. They were transferred onto a hot plate for hot digestion which went on until brown fumes were given off from the samples. Then the contents were washed down using distilled water and left to boil to below 50 mL. The digests were cooled and filtered through Whatmann filter papers. The filtrates were transferred into 100 mL volumetric flasks and made up to the mark with deionized water.

The determination of concentrations of HeMs was conducted using a Perkin Elmer atomic absorption spectrophotometer (AAS, Analyst 100) at 248.3, 279.5, 213.9, 324.7, 232.0, 228.8, 248.3, 238.3 and 357.9 nm for Fe, Mn, Zn, Cd, Pb, Cr, Cu, Co and Ni, respectively [34,35].

The elemental concentrations were established from calibration curves constructed from diluted working standards of 1000 ppm stock solutions of nitrate and chloride salts of the HeMs. The linearity of the curves fell within acceptable limits (R^2^ > 0.996). The assurance of the quality of the analytical results was addressed through analysis of procedural blanks and spiked samples, whose recoveries were found to be acceptable (range: 98% to 100%). The relative standard deviations of the experiments (analytical precision) varied from 3.5 to 5.0%.

### 2.5. Human Health Risk Assessment Models

The carcinogenic and non-cancer health risk assessments involved the computation of average daily dose (*ADD*), hazard quotient, hazard index and cancer risk for both adults and children [13]. The *ADD* (mg/kg/day) was estimated to discern human exposure through direct ingestion of maize flour as bread or porridge (Equation (1)).

(1) $$\mathrm{ADD}=\frac{C\times IR\times E_{f}\times E_{d}}{W_{ab}\times T_{aet}}\;$$

where *C* is the concentration of the heavy metal (mg/kg), *IR* is the ingestion rate = 100 mg/kg/day and 200 mg/kg/day for children and adults, respectively, *Ef* is the exposure frequency = 365 days/year, *Ed* is the exposure duration = 58.65 years for an adult Ugandan [36,37] and W*ab* is the average body weight; as per the clinical guidelines from the Ugandan government, children are those who are 6 years of age weighing 15 kg, while adults were defined as those who are 30 years or older weighing 70 kg [38], and *Tae* is average exposure time for non-carcinogens = *Ef* × *Ed.*

The hazard quotient (HQ) was calculated to establish non-cancer risks from the HeMs (Equation (2)). Since the effects of HeMs can be augmentative, the hazard index (HI) or total HQ was consequently calculated (Equation (3)).

(2) $$\mathrm{HQ}=\frac{ADD}{R_{f}D}$$

(3) HI=∑HQ

where *R_f_D* is the oral reference dose = 7.0 × 10^–1^, 1.4 × 10^−1^, 3.0 × 10^−1^, 1.0 × 10^−3^, 3.5 × 10^–3^, 1.5 × 10^0^, 2.0 × 10^−2^ and 2.0 × 10^−2^ mg/kg/day for Fe, Mn, Zn, Cd, Pb, Cr, Cu, Co and Ni, respectively as per the US EPA [13,39,40,41,42].

The carcinogenic health risk (CR) was computed as the incremental lifetime cancer risk for the carcinogenic elements (Pb, Ni, Cr and Cd) (Equation (4)). The total cancer risk (TCR) was estimated as the sum of all the cancer risks due to the ingestion of the four heavy metals in maize flour (Equation (5)).

CR = *ADD* × CSF (4)

(5) $${\mathrm{TCR}}_{\;}=\sum_{1}^{4}CR$$

where CSF is the ingestion cancer slope factor = 8.5, 0.91, 0.5 and 6.1 for Pb, Ni, Cr and Cd mg/kg-day, respectively [43]. The CR estimates the probability of the cancerogenic effect, and values higher than 10^−4^ indicate unacceptable risks.

### 2.6. Statistical Analysis

Quantitative data from experimental analyses performed in triplicate were captured in Microsoft Excel (Microsoft Corporation, Redmond, WA, USA) where they were averaged and expressed as means ± standard deviations of replicates. The concentration of the HeMs was compared with the Ugandan standard for maize flour (US EAS 44: 2019) [44], WHO/FAO [45] and those of Codex Alimentarius Commission [46,47]. Significant differences in the mean concentration of the HeMs were established using one-way analysis of variance, followed by Tukey posthoc test. The analyses were accomplished at *p* < 0.05 using GraphPad Prism for Windows (v9.3.1, GraphPad software, San Diego, CA, USA).

## 3. Results

### 3.1. Deposition and Distribution of Toxic Elements in the Samples by Processing Method

The effect of different mechanical grinding methods on the deposition and concentrations of some selected HeMs in milled maize from Mbarara city, Uganda was investigated. The concentrations of HeMs in maize flour samples were higher in those ground using the metallic mortar than the wooden mortar (*p* < 0.05). The results indicate that the contribution of the metallic mortar was highest with regard to the HeMs Fe, Mn, Zn, Pb and Cu as compared to the other metals (Table 1). Thus, the metallic mortar adds reasonable amounts of these metals if it is used as a method of maize milling. The wooden mortar added more amounts of Fe and Zn (*p* < 0.05) when compared to the other elements determined. Nevertheless, the concentration of Cd, Pb, Cr, Co and Ni were found to be higher than the WHO/FAO food standards [45] (Table 1). However, this method produced traces of Cd, Cr, Co and Ni.

On the other hand, maize flour obtained using hammer mills had higher levels of HeMs than that in samples from the wooden mortar (*p* < 0.05). Samples milled using electric motor−powered mills had higher HeMs than those produced using diesel engine−powered mills. However, these differences were not statistically significant (*p* < 0.05). In comparison to the Ugandan standard (US EAS 44:2019) [44], the heavy metal content of the flours surpassed the regulatory guidelines for Pb. Further comparison with international guidelines showed that with the exception of Ni, Zn and Cr, most samples had HeMs (Fe, Pb and Cd) in levels which exceeded the permissible levels. Taken together, the obtained results suggest that the local community of Mbarara city is exposed to potential effects of HeMs, especially for those who consume maize flours processed using metallic mortars and some commercial hammer mills.

### 3.2. Non-Carcinogenic Health Risk Assessment Results

**Figure 3:**
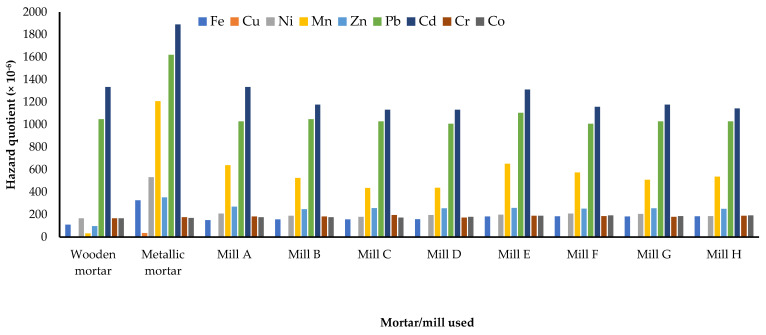
Mean hazard quotients for ingestion of heavy metals in milled maize by children in Mbarara City, Western Uganda. Mills A to D were diesel engine−powered while E to H were electric motor−powered.

**Figure 4:**
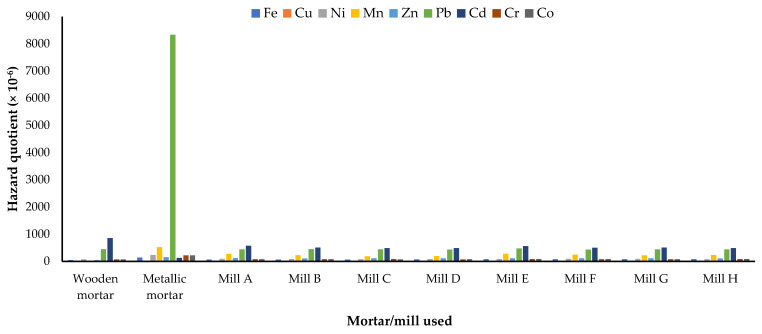
Mean hazard quotients for ingestion of heavy metals in milled maize by adults in Mbarara City, Western Uganda. Mills A to D were diesel engine−powered while E to H were electric motor−powered.

**Figure 5:**
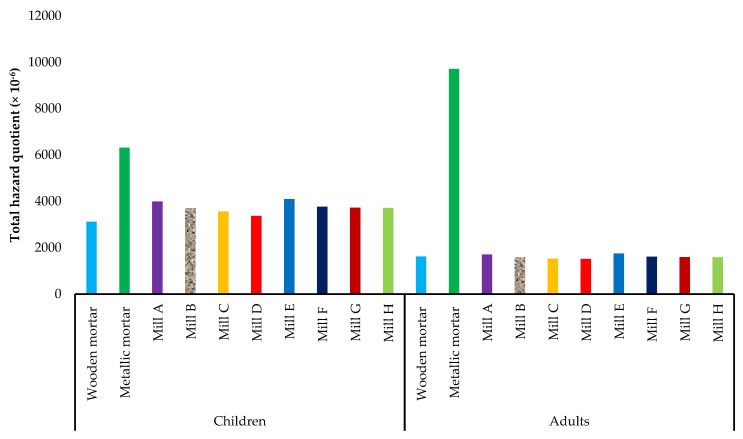
Total hazard quotients for ingestion of heavy metals in milled maize by children and adults in Mbarara City, Western Uganda. Mills A to D were diesel engine−powered while E to H were electric motor−powered.

The daily dose through ingestion spans from 1.42 × 10^−6^ mg kg^−1^ day^−1^ for Cu and Ni ingested in maize milled in wooden mortar by adults to 229.67 × 10^−6^ mg kg^−1^ day^−1^ for Fe in maize flour milled in a metallic mortar when consumed by children (Table 2). In children, the hazard quotient ranged from 2.20 × 10^−6^ (for Cu from maize flour milled by use of the wooden mortar) to 1890 × 10^−6^ for Cd in maize milled with the metallic mortar. For adults, the hazard quotients span from 0.95 × 10^−6^ (for Cu from maize flour milled by use of the wooden mortar) to 8325.71 × 10^−6^ for Pb in maize milled with the metallic mortar. Accordingly, the hazard quotients from Pb and Cd, as well as the total hazard quotient, were less than 1, showing that there are no potential health risks from the ingestion of milled maize flour (Figure 3, Figure 4 and Figure 5). However, Pb and Cd are the major contributors to the non-carcinogenic effects that could arise from the consumption of maize flours obtained through the different mechanical milling methods.

### 3.3. Cancer Health Risk Assessment Results

For the incremental life cancer risks, the risk values of the individual groups were computed (Table 3). For the children, the values ranged from 1.665 × 10^−6^ for Cr in flour milled using a wooden mortar to 48.195 × 10^−6^ for Pb in flour milled using a metallic mortar. For children as a sensitive group, the individual metal and tPlease use commas to separate thousands for numbers with five or more digits (not four digits) in the picture, e.g., “10000” should be “10,000”.otal cancer risk values were within the acceptable range of 1 × 10^−6^ to 1 × 10^−4^, implying that there are no potential carcinogenic health risks for children who consume the maize flour. For adults, cancer risk values ranged from 0.715 × 10^−6^ for Cr to 247.690 × 10^−6^ for Pb. In adults, the individual cancer risk value for Pb in flour milled using a metallic mortar was greater than the safe limit of 1 × 10^−6^ to 1 × 10^−4^.

## 4. Discussion

Nutritional food quality plays an important role in maintaining human health. However, food and drinking water, along with occupational exposure, are the main routes of exposure to potentially toxic elements in humans. In this study, the concentrations of HeMs in milled maize flours were higher in samples obtained using the metallic mortar than the wooden mortar. However, relatively higher levels of Fe and Zn leached from the wooden mortar into the flour probably because the wooden mortar and pestle are made of natural solid materials which contain phytoavailable levels of geogenic HeMs. The highest concentrations of HeMs were recorded in flour samples obtained using the metallic mortar, though Cd, Cr, Co and Ni occurred as traces. The high levels may be attributed to the presence of these metals as impurities which are added or not removed during metal refining processes. In Uganda, metallic mortars are shared within the neighborhood for preparing small quantities of food and medicinal herbs which cannot be commercially milled. These metallic mortars are possible sources of food contaminants because they are fabricated from rusty and corroded metallic pipes that have been discarded from the Kilembe mines (Western Uganda) and later collected by scrap dealers for recycling. Without any tests for hardness and other chemical properties, local artisans fabricate these pipes into food equipment for use in small scale food industries.

For the maize flour obtained using hammer mills, the higher levels of HeMs than that found in samples from the wooden mortar could be because hammer mills are fabricated using mild steel which contains Fe, Mn, Cr and Cu [48]. Depending on the concentration of the HeMs in the plates, the variations in the HeMs content should not vary greatly between the electric motor− and diesel engine−powered mills. The concentrations of HeMs in milled maize from Mbarara City were higher than reported in some previous studies in Kampala (Uganda) [33] and the Tolon District (Ghana) [13] (Table 4). Another study in the Accra metropolis (Ghana) [14] reported higher levels of Ni (26.18–46.42 mg/kg) and Cd (4.80–6.40 mg/kg) in mechanically milled maize flour than was found in this study. Oniya et al. [16] found very high concentrations of Fe (50–368 mg/kg), Zn (16.0–22.0 mg/kg) and Cr (2.0–14.0 mg/kg) in maize flour milled using hammer mills in Ondo State (Nigeria). Similarly, Lebnebiso et al. [17], Nnaji et al. [21] and Kalagbor et al. [18] found elevated levels of Fe (72.1–318.2 mg/kg, 270.34–327.49 mg/kg and 16.75–43.00 mg/kg) in maize flours milled using hammer mills in the Jimeta Modern Market (Nigeria), the Ubani, Orie Ugba and Ubakala Community Markets (Nigeria), and Port Harcourt (Nigeria). Ofori et al. [20] found Fe (20.44 mg/kg) in maize flour milled using hammer mills in Accra (Ghana) which is comparable to the levels quantified in this investigation. However, they found a higher level of Zn (6.04 mg/kg) than was found in this study. The differences in the levels of HeMs in maize flours as compared with our study could be due to differences in the levels of metals that leached into the flour during milling, initial metal accumulation in the grains during plant growth and the use status of the mill plates at the time of milling.

In the human health risk assessment, the results of our study agreed with a previous study done in Kampala (Uganda) [33] in which the calculated health risks suggested that consumers of maize flour produced using hammer mills are not likely to experience adverse non-carcinogenic health effects. Larson et al. [13] also indicated that no potential hazards could be associated with children’s and adult’s dietary intake of HeMs leached into maize flour milled using some local mills in Ghana. In a similar study [21] examining potential HeM enrichment in flour milled using hammer mills with locally fabricated diesel engine−powered metallic disc grinders in Umuahia (Nigeria), it was found that the health risk index for Pb and Cr in maize exposed the consuming population to some non-carcinogenic health risks.

For the cancer risk assessment, the individual and total cancer risk values for Pb in flour milled using the metallic mortar surpassed the safe limit suggested by US EPA, suggesting potential cancer risks in the exposed population. A similar study [21] of maize flour milled using hammer mills in Umuahia (Nigeria) reported that the carcinogenic risk values for Ni and Cr exceeded the 1 × 10^−4^ threshold provided by the US EPA. Another study in Ghana [13] concluded that no potential cancer risks could arise from the intake of HeMs deposited into maize flour during hammer-type mechanical milling. These results indicate that there is a need to embrace diet diversity, especially in instances where maize is processed using a traditional metallic mortar.

## 5. Conclusions

This study revealed that the milling method used for processing maize into flour had an effect on (enriched) the heavy metal content of the flours. The metals were plausibly deposited into the flour due to frictional wear of the mortars/mill plates during milling. The concentrations of the HeMs in maize flour produced by metallic mortar and commercial mills were found to be higher than the dietary tolerance limits set by the Uganda National Bureau of Standards (for Pb) and the WHO/FAO (for Fe, Pb and Cd). Human health risk assessment showed that there are potential carcinogenic health risks from adults’ intake of HeMs in maize flour milled using a metallic mortar. Thus, the processing of maize flour needs to be monitored by the relevant government bodies such as UNBS which are mandated to control quality manufacturing (fabrication) of food processing equipment including mill plates to minimize the possibilities of heavy metal contamination of human food and animal feed.

## Figures and Tables

**Table 1:** Heavy metal concentration (mg/kg) in milled maize flours from Mbarara city, Uganda obtained using different mechanical milling methods.

Mortar/Mill	Fe	Cu	Ni	Mn	Zn	Pb	Cd	Cr	Co
Wooden mortar	11.60 ± 0.04	0.50 ± 0.07	0.50 ± 0.00	0.70 ± 0.01	4.40 ± 0.05	0.55 ± 0.01	0.60 ± 0.08	0.50 ± 0.02	0.50 ± 0.00
Metallic mortar	34.45 ± 0.95	8.10 ± 0.07	1.60 ± 0.10	25.40 ± 0.03	15.90 ± 0.08	10.20 ± 0.00	0.85 ± 0.01	1.53 ± 0.95	1.51 ± 0.06
Mill A	15.80 ± 2.17	0.68 ± 0.29	0.63 ± 0.15	13.43 ± 3.60	12.20 ± 1.48	0.54 ± 0.07	0.60 ± 0.09	0.55 ± 0.09	0.53 ± 0.09
Mill B	16.51 ± 1.12	0.58 ± 0.23	0.57 ± 0.11	11.05 ± 3.43	11.17 ± 1.11	0.55 ± 0.12	0.53 ± 0.06	0.55 ± 0.10	0.53 ± 0.06
Mill C	16.48 ± 0.79	0.81 ± 0.53	0.56 ± 0.09	9.18 ± 3.98	11.60 ± 0.64	0.54 ± 0.08	0.51 ± 0.03	0.59 ± 0.09	0.52 ± 0.04
Mill D	16.73 ± 0.86	0.63 ± 0.29	0.59 ± 0.10	9.23 ± 3.60	11.53 ± 0.57	0.53 ± 0.09	0.51 ± 0.03	0.52 ± 0.04	0.54 ± 0.07
Mill E	19.33 ± 2.51	0.66 ± 0.34	0.60 ± 0.12	13.71 ± 4.65	11.68 ± 1.25	0.58 ± 0.08	0.59 ± 0.11	0.57 ± 0.14	0.57 ± 0.08
Mill F	19.39 ± 2.58	0.52 ± 0.04	0.63 ± 0.15	12.08 ± 1.21	11.35 ± 0.49	0.53 ± 0.07	0.52 ± 0.04	0.56 ± 0.09	0.58 ± 0.10
Mill G	19.23 ± 1.55	0.58 ± 0.29	0.62 ± 0.15	10.70 ± 0.39	11.53 ± 0.50	0.54 ± 0.07	0.53 ± 0.05	0.54 ± 0.12	0.56 ± 0.12
Mill H	19.37 ± 0.40	0.61 ± 0.38	0.56 ± 0.10	11.28 ± 0.80	11.32 ± 0.84	0.54 ± 0.07	0.51 ± 0.03	0.57 ± 0.09	0.58 ± 0.08
National standard (US EAS 44:2019) [44]	—	—	—	—	—	0.20	0.10	—	—
International guidelines [45,46,47]	15.0	40.0	10.0	2.3	30.0	0.2	0.3–0.7	1.3	—

Note: Results are presented as means ± standard deviation of 10 analyses—means not indicated. Mills A to D were diesel engine-powered while E to H were electric motor-powered.

**Table 2:** Average daily dose (×10^−6^ mg/kg/day) of milled maize flours from Mbarara city, Uganda obtained using different mechanical milling methods.

Age Group	Mortar/Mill	Fe	Cu	Ni	Mn	Zn	Pb	Cd	Cr	Co
Children	Wooden mortar	77.33	3.33	3.33	4.67	29.34	3.67	4.00	3.33	3.33
	Metallic mortar	229.67	54.00	10.67	169.34	106.00	5.67	5.67	3.53	3.40
	Mill A	105.34	4.53	4.20	89.54	81.34	3.60	4.00	3.67	3.53
	Mill B	110.53	3.87	3.80	73.67	74.47	3.67	3.53	3.67	3.53
	Mill C	109.87	5.40	3.60	61.20	77.34	3.60	3.40	3.93	3.47
	Mill D	111.53	4.20	3.93	61.53	76.87	3.53	3.40	3.47	3.60
	Mill E	128.87	4.40	4.00	91.40	77.87	3.87	3.93	3.80	3.80
	Mill F	129.27	3.47	4.20	80.53	75.67	3.53	3.47	3.73	3.87
	Mill G	128.20	3.87	4.13	71.34	76.87	3.60	3.53	3.60	3.73
	Mill H	129.13	4.07	3.73	75.20	75.47	3.60	3.43	3.80	3.87
Adults	Wooden mortar	33.14	1.42	1.42	2.00	12.57	1.57	1.71	1.43	1.43
	Metallic mortar	98.43	23.1	4.57	72.57	45.42	29.14	2.43	4.37	4.31
	Mill A	45.14	1.94	1.80	38.37	34.86	1.54	1.71	1.57	1.51
	Mill B	47.17	1.66	1.63	31.57	31.91	1.57	1.51	1.57	1.51
	Mill C	47.08	2.31	1.60	26.23	33.14	1.54	1.46	1.68	1.49
	Mill D	47.80	1.80	1.68	26.37	32.94	1.51	1.46	1.49	1.54
	Mill E	55.23	1.88	1.71	39.17	33.37	1.66	1.68	1.63	1.63
	Mill F	55.40	1.49	1.80	34.05	32.43	1.51	1.49	160	1.66
	Mill G	54.94	1.66	1.77	30.57	32.94	1.54	1.51	1.54	1.60
	Mill H	55.34	1.74	1.60	32.23	32.34	1.54	1.46	1.63	1.66

Mills A to D were diesel engine-powered while E to H were electric motor-powered.

**Table 3:** Cancer risk values from consumption of milled maize flours in Mbarara city, Uganda obtained using different mechanical milling methods.

Age Group	Mortar/Mill	Cancer Risk Value (×10^−6^)				Total Cancer Risk (×10^−6^)
		Pb	Ni	Cr	Cd	
Children	Wooden mortar	31.195	3.030	1.665	24.400	60.290
	Metallic mortar	48.195	9.710	1.765	34.587	94.257
	Mill A	31.195	3.822	1.835	24.400	61.270
	Mill B	31.195	3.458	1.835	21.533	58.039
	Mill C	30.600	3.276	1.965	20.740	56.581
	Mill D	30.005	3.576	1.735	20.740	56.056
	Mill E	32.895	3.640	1.900	23.973	62.408
	Mill F	30.005	3.822	1.865	21.167	56.859
	Mill G	30.600	3.758	1.800	21.533	57.691
	Mill H	30.600	3.394	1.900	20.923	56.824
Adults	Wooden mortar	13.345	1.292	0.715	10.431	25.783
	Metallic mortar	**247.690** ^1^	4.158	2.185	14.823	**268.856** ^1^
	Mill A	13.090	1.638	0.785	10.431	25.944
	Mill B	13.345	1.483	0.785	9.211	24.824
	Mill C	13.090	1.456	0.840	8.906	24.292
	Mill D	12.835	1.529	0.745	8.906	24.015
	Mill E	14.110	1.556	0.815	10.248	26.729
	Mill F	12.835	1.638	0.800	9.089	24.362
	Mill G	13.090	1.611	0.770	9.211	24.682
	Mill H	13.090	1.456	0.815	8.906	24.267

^1^ Boldened values are outside the US EPA safe limit of 1 × 10^−6^ to 1 × 10^−4^.

**Table 4:** Comparison of heavy metal concentrations (mg/kg) in commercially milled maize flours from Mbarara city (Uganda) with other global reports.

Area (Country)	Fe	Cu	Ni	Mn	Zn	Pb	Cd	Cr	Co	References
Mbarara (Uganda)	11.60–34.45	0.50–8.10	0.50–1.60	0.70–25.40	4.40–15.90	0.53–10.20	0.51–0.85	0.50–1.53	0.50–1.51	This study
Kampala (Uganda)	0.257–1.782	0.016–0.198	—	BDL–1.151	—	—	—	0.122–0.501	—	Ainebyona [33]
Accra metropolis (Ghana)	—	0.70–1.50	26.18–46.42	1.35–4.10	0.52–0.90	—	4.80–6.40	—	—	Abrefah et al. [14]
Tolon District (Ghana)	1.3392	—	0.9502	0.3550	0.6809	2.2177	—	0.4359	—	Larsen et al. [13]
Markets in Umuahia (Nigeria)	270.34–636.78	—	BDL	—	—	BDL—2.75	—	4.8–12.6	—	Nnaji et al. [21]
Ondo State (Nigeria)	50–368	BDL–2.0	—	—	16.0–22.0	BDL	BDL	2.0–14.0	—	Oniya et al. [16]
Jimeta Modern Market (Nigeria)	72.1–318.2	—	—	—	—	—	—	—	—	Lebnebiso et al. [17]
Port Harcourt (Nigeria)	16.75–43.00	—	—	—	—	—	—	—	—	Kalagbor et al. [18]
Accra (Ghana)	20.44	0.03	—	—	6.04	<0.01	—	—	—	Ofori et al. [20]

BDL = Below Method Detection Limit; CAC = Codex Alimentarius Commission; —means Not Determined.

## Data Availability

Data supporting the conclusions of this study are available on request from the authors.
